# Acute pulmonary edema caused by takotsubo cardiomyopathy in a pregnant woman undergoing transvaginal cervical cerclage: A case report

**DOI:** 10.1097/MD.0000000000005536

**Published:** 2017-01-10

**Authors:** Jae-Young Lee, Hyun-Jung Kwon, Sang-Wook Park, Yu-Mi Lee

**Affiliations:** Department of Anesthesiology and Pain Medicine, Asan Medical Center, University of Ulsan College of Medicine, Seoul, Republic of Korea.

**Keywords:** pregnant woman, pulmonary edema, takotsubo cardiomyopathy

## Abstract

**Background:**

The physiological changes associated with pregnancy may predispose pregnant women to pulmonary edema. Other known causes of pulmonary edema during pregnancy include tocolytic drugs, preeclampsia, eclampsia, and peripartum cardiomyopathy.

**Methods:**

We describe a rare case of pulmonary edema caused by takotsubo cardiomyopathy in a pregnant woman at 14 weeks of gestation who was undergoing emergency transvaginal cervical cerclage.

**Results:**

Intraoperative chest radiography revealed severe pulmonary edema and echocardiography indicated moderate left ventricular dysfunction with akinesia of the mid to apical left ventricular wall segment, which is reflective of takotsubo cardiomyopathy.

**Conclusion:**

With early detection and appropriate management, the patient was stabilized in a relatively short period of time. Based on her clinical signs and symptoms, we suspect that the pulmonary edema was caused by takotsubo cardiomyopathy.

## 1 Introduction

Pulmonary edema is characterized by the accumulation of fluid in the pulmonary interstitial spaces and alveoli that prevents the adequate diffusion of both oxygen and carbon dioxide.^[1]^ Pulmonary edema development is more likely in pregnant women due to the physiological changes associated with pregnancy. Other known causes of pulmonary edema during pregnancy include simultaneous use of multiple tocolytics, infection, pre-existing cardiac disease, preeclampsia, and eclampsia.^[1]^ Pulmonary edema development during pregnancy is associated with an increased risk of maternal and fetal morbidity and mortality.^[2]^ In the present report, we describe a rare case of acute pulmonary edema that was likely caused by takotsubo cardiomyopathy in a pregnant woman. We believe that favorable outcomes can be ensured in such cases by increased awareness of cardiogenic pulmonary edema, early identification of the condition, and administration of appropriate treatment.

## 2 Case report

**Figure 1:**
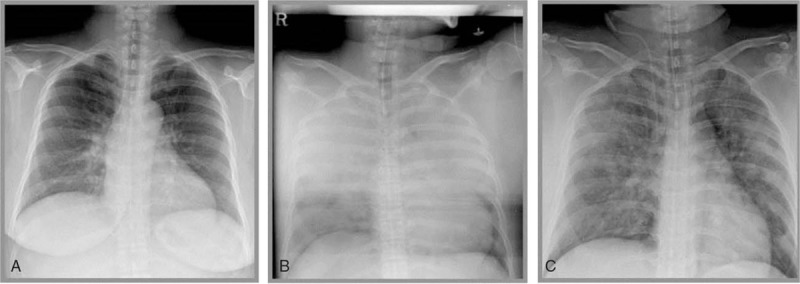
Changes in the chest radiograph findings. (A) Preoperative chest radiograph at 14 days prior to surgery. (B) Intraoperative chest radiograph. Opacification at the bilateral lung fields was notable, suggesting pulmonary edema. (C) Chest radiograph on postoperative day 1. Resolution of the pulmonary edema can be observed.

A 32-year-old woman (gravida 2, para 1) was diagnosed with an incompetent cervix at 14 weeks of gestation and was scheduled to undergo emergency transvaginal cervical cerclage. She did not have any relevant medical history. She had undergone an elective cesarean delivery 3 years before. The patient's height and weight were 154 cm and 81 kg, respectively. Before admission, she was taking a 2-month course of herbal medication. After taking the medication, she gained 7 kg in the week prior to admission. After admission, she was given ritodrine to treat the incompetent cervix. The preoperative laboratory evaluation findings, including chest radiography (14 days prior to surgery), were not remarkable (Fig. 1A). Regarding her preoperative vital signs, her blood pressure (BP) was 100/77 mm Hg, heart rate was 100 beats/min (regular sinus rhythm), and peripheral oxygen saturation (SpO_2_) was 95%. The surgery was performed with the patient in the Trendelenburg position and under local anesthesia. However, at that point, she complained of dyspnea and pain at the operative site. The severity of the dyspnea made it impossible to maintain the Trendelenburg position and, hence, she was placed in a head-up position. Ten minutes after surgery, she developed shortness of breath and exhibited a poor pulse oximetry waveform. Her BP was 110/70 mm Hg. However, her heart rate measured by electrocardiogram (ECG) and SpO_2_ could not be monitored due to her agitation. The surgeon required assistance from the anesthesiologist to rapidly resolve the problem.

Accordingly, the patient was monitored via noninvasive BP determination, ECG, and pulse oximetry. Anesthesia was intravenously induced using thiopental sodium (250 mg) and succinylcholine (75 mg). The trachea was promptly intubated while Sellick maneuver was performed to prevent aspiration. Anesthesia was maintained using 2 L/min O_2_, 2 L/min air, and 1 to 2 vol% sevoflurane (inspired). Volume-controlled mechanical ventilation was performed with a tidal volume of 500 mL, positive end-expiratory pressure of 5 cm H_2_O, and respiratory rate of 12 to 15 breaths/min to maintain an end-tidal carbon dioxide concentration of 32 to 35 mm Hg. The SpO_2_ decreased rapidly to as low as 90% immediately after the tracheal intubation. The surgery was quickly completed, but she exhibited new-onset audible crackles in both lung fields. On endotracheal suction, large amounts of pink frothy foamy fluid were drained. The patient's condition did not improve. Hence, the delivery of anesthetics was discontinued and 100% O_2_ (6 L/min) was administered. She exhibited spasmodic coughing and a consistent discharge of pink frothy sputum, along with the extrusion of large amounts of foamy fluid. Auscultation revealed crackles bilaterally in both lung fields. The SpO_2_ further decreased to 80% after 1 min, and the systolic BP decreased to 80 mm Hg. Intraoperative chest radiography indicated increased opacification of the bilateral lung fields, which suggested severe pulmonary edema (Fig. 1B). She was treated with an injection of furosemide (20 mg). In addition, an arterial line was placed into the right radial artery to continuously monitor BP and collect blood samples and a central venous catheter was placed into the right internal jugular vein to monitor central venous pressure and administer drugs. The total amount of infused intravenous fluids during the surgery was 500 mL of lactated Ringer solution. Arterial blood gas analysis exhibited a pH of 6.99, PaCO_2_ of 101 mm Hg, PaO_2_ of 51 mm Hg, and SaO_2_ of 63% (Table 1). Ventilation was increased to correct the hypoxia and hypercapnia. In addition, dobutamine (5 μg/kg per min) and norepinephrine (0.05 μg/kg per min) were intravenously administered. At 30 min after tracheal intubation, the SpO_2_ gradually increased to 90%.

**Table 1:**
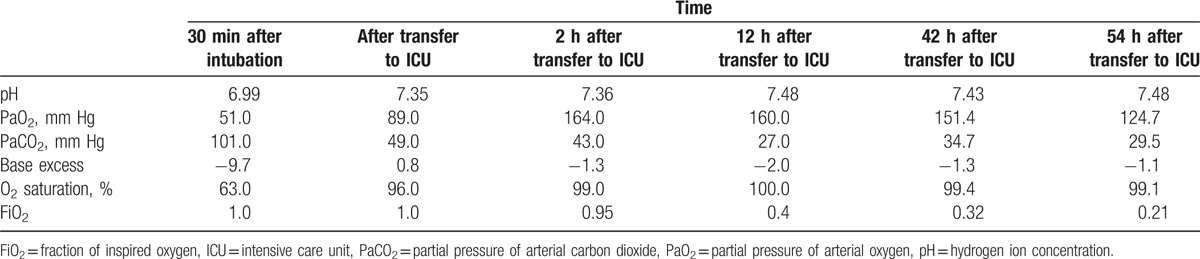
Serial changes in arterial blood gas analysis data.

**Figure 2:**
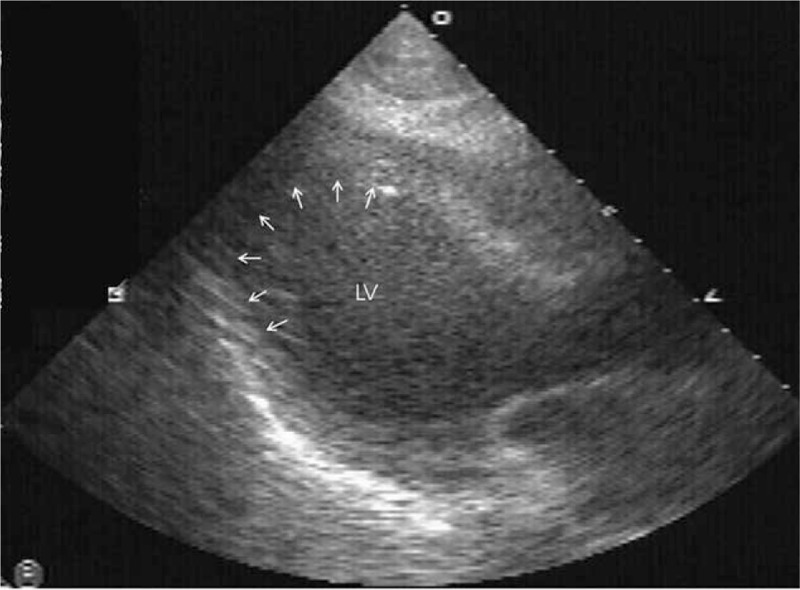
Portable transthoracic echocardiograph (parasternal long axis view) showing typical ballooning of the apex in the heart during systole. LV = left ventricle.

Her ECG showed sinus tachycardia without ST-T wave change. Intraoperative transthoracic echocardiography, performed by an anesthesiologist, exhibited an ejection fraction of 40% and akinesia of the mid to apical left ventricular (LV) wall segments (Fig. 2).

She was transferred to the intensive care unit with endotracheal intubation. She still exhibited hypotension and an oliguric state. Continuous renal replacement therapy was therefore conducted along with intravenous infusion of dobutamine and norepinephrine to rapidly resolve the pulmonary congestion. The levels of cardiac troponin I (1.135 ng/mL; normal range, <1.5 ng/mL) and creatine kinase MB (3.8 ng/mL; normal range, <5 ng/mL) were somewhat elevated, although both remained within normal limits.

On postoperative day (POD) 1, her vital signs were stable and there were no signs of respiratory disturbance. Moreover, no crackles were audible on auscultation. Follow-up chest radiography showed a decrease in the pulmonary congestion (Fig. 1C). Therefore, extubation was performed and treatment with inotropic agents, vasopressors, and continuous renal replacement therapy was discontinued. She was transferred to the general ward in an overall stable condition while the fetal status was continuously observed via obstetric ultrasonography. On POD 3, follow-up echocardiography revealed that the ejection fraction had improved to 52%, with normal LV wall motion. On POD 13, she was discharged, with no complications noted in the patient or fetus.

## 3 Discussion

Information on the natural history of pulmonary edema in pregnant women is scarce. Due to the presence of a reduced plasma protein osmotic pressure, even small changes in hydrostatic pressure can lead to pulmonary edema. These physiological changes could explain the development of pulmonary edema in pregnant women with normal or minimally elevated pulmonary capillary wedge pressure.^[2]^ In fact, pulmonary edema is reported to develop in 0.05% of the general obstetric population.^[2]^ The potential cause of the pulmonary edema should be determined to identify the specific therapy required to reverse the primary pathological process. In the present case, the potential causes of pulmonary edema included peripartum cardiomyopathy, use of tocolytics, and stress-induced cardiomyopathy.

The acute and rapidly reversible LV failure observed in the present case corresponds to peripartum cardiomyopathy with the following typical elements: pulmonary edema, echocardiographic LV dysfunction, and no other cause of cardiomyopathy or myocarditis. This condition can be diagnosed only if the other causes of cardiomyopathy are absent. The diagnostic criteria for peripartum cardiomyopathy include cardiac failure development in the last month of pregnancy or within 5 months of delivery; no identifiable cause of the cardiac failure; no recognizable heart disease before the last month of pregnancy; and an ejection fraction of <45% or a combination of an M-mode fractional shortening of <30% and an end-diastolic dimension of >2.7 cm/m^2^.^[3,4]^

The symptoms of heart failure, such as dyspnea, dizziness, pedal edema, and orthopnea, can also develop in patients with normal pregnancy.^[3]^ Hence, pregnant women with peripartum cardiomyopathy may consider their symptoms to be normal. However, the sudden development of swelling and other heart failure symptoms in an otherwise normal case of pregnancy in any peripartum patient should prompt physicians to consider peripartum cardiomyopathy. In the present case, because the patient was only at 14 weeks of gestation, we believed that it was inappropriate to diagnose the patient with peripartum cardiomyopathy.

Ritodrine and terbutaline are the 2 most common β_2_-sympathomimetic agents used for tocolysis.^[5]^ Tocolytic-induced pulmonary edema is a unique syndrome that develops as a complication of sympathomimetic therapy for premature labor. Dyspnea, chest pain, and cough are the major symptoms of tocolytic-induced pulmonary edema, and physical examination in such cases typically indicates tachypnea and tachycardia while auscultation reveals bibasilar crackles without any clinical evidence of heart failure.^[5]^ In our present case, the patient was also treated with ritodrine. However, the echocardiographic findings in tocolytic-induced pulmonary edema are usually normal, inconsistent with the present case.

Takotsubo cardiomyopathy, apical ballooning syndrome, or stress cardiomyopathy have recently been reported as conditions that develop from a transient stunned myocardium as a result of psychological and/or physical stress.^[1]^ In 1991, Dote et al^[6]^ first described a syndrome characterized by transient wall motion abnormalities that extended beyond a single coronary distribution and involved the apical and mid-portions of the left ventricle in the absence of significant obstructive coronary disease. The syndrome was initially described in Japan as “takotsubo” due to the similarity in its shape to that of a Japanese octopus pot. The pathophysiological mechanism underlying the syndrome remains unclear, although exaggerated sympathetic stimulation is believed to be critically involved.^[7]^ This condition appears to predominantly develop in postmenopausal women.^[8]^ However, a case of takotsubo cardiomyopathy in an eclamptic pregnant patient was reported recently by Kunal et al.^[9]^ The most commonly reported cardinal presenting symptoms include chest pain and dyspnea, although initial presentations with syncope and palpitations, nausea and vomiting, hypotension and shock, ventricular fibrillation, or cardiac arrest have also been reported.^[10]^

Takotsubo cardiomyopathy is diagnosed based on the presence of Mayo clinic criteria at presentation; that is, transient hypokinesis, akinesis, or dyskinesis of the LV mid-segments, with or without apical involvement; regional wall motion abnormalities extending beyond a single epicardial vascular distribution; and, often (but not always), presence of a stressful trigger; absence of obstructive coronary disease or angiographic evidence of acute plaque rupture; new ECG abnormalities (either ST-segment elevation and/or T wave inversion) or modest elevation of cardiac troponin levels; and absence of pheochromocytoma and myocarditis. For the diagnosis of takotsubo cardiomyopathy, all of these 4 criteria must be present.^[11]^

In the present case, a young woman in her first trimester of pregnancy presented with dyspnea at onset, prior to the induction of general anesthesia for emergency surgery. However, there were some limitations for the diagnosis of takotsubo cardiomyopathy, including the lack of changes in the ECG and ST-T wave and normal cardiac enzyme test results (despite some elevation). Nevertheless, she exhibited some other characteristics commonly observed in takotsubo cardiomyopathy, including pulmonary edema, moderate LV dysfunction, and the echocardiographic features of this condition. The patient quickly recovered normal LV function, which is typical in this syndrome and indicates its reversibility. According to Grayburn and Hill,^[12]^ significant operative pain, withdrawal of anesthesia, or shifts in intravascular volume may contribute to cardiac events in patients undergoing noncardiac surgery. In the present case, the perioperative stress, such as pain, anxiety, and respiratory discomfort, caused by the Trendelenburg position could have contributed to the development of takotsubo cardiomyopathy, and the intraoperative administration of catecholamines and vasoconstrictive substances may have enhanced the cardiopulmonary compromise.

The treatment of pulmonary edema depends on several basic principles, independent of etiology. Most cases of pulmonary edema exhibit some response to diuresis, regardless of the etiology.^[2]^ Nevertheless, the diagnostic measures are very important. After the initial stabilization of the condition, steps should be taken to accurately determine the specific pathology involved. Historical factors may help to elucidate the cause.^[2]^

In most cases of pulmonary edema in pregnant women, the symptoms resolve with the judicious use of diuretics, supplemental oxygen, and restriction of fluid administration. Despite such measures, the symptoms progress in approximately 15% to 20% of patients, who then require intubation and mechanical ventilation.^[5]^ Both the diagnosis of pulmonary edema and the understanding of the underlying pathophysiology have important implications for treatment. Moreover, a registry of acute pulmonary edema should be established to investigate its natural history. We believe that the development of this syndrome should be anticipated in pregnant woman, and the condition should be urgently treated to prevent further respiratory and cardiovascular compromise.

## 4 Informed consent

This study adhered to the tenets of the Declaration of Helsinki. The patient signed informed consent for the publication of this case report and any associated images.
